# Granular Cell Tumor of the Dorsal Tongue in a 58-Year-Old Woman: A Case Report

**DOI:** 10.7759/cureus.91577

**Published:** 2025-09-03

**Authors:** Athanasios Vlachodimitropoulos, Georgios Batsaouras, Spyridon Lygeros, Vasileios Brestas, Vasiliki Tzelepi

**Affiliations:** 1 Otolaryngology - Head and Neck Surgery, University General Hospital of Patras, Patras, GRC; 2 Pathology, University General Hospital of Patras, Patras, GRC

**Keywords:** abrikossoff’s tumor, benign oral tumor, granular cell tumor, oral pathology, pseudoepitheliomatous hyperplasia, schwann cell tumor, tongue neoplasm

## Abstract

Granular cell tumor (GCT) is a rare, typically benign neoplasm of Schwann cell origin, most commonly affecting the oral cavity and particularly the tongue. We report a case of a 58-year-old woman who presented with a painless, slow-growing submucosal nodule on the dorsal surface of the tongue. Clinical examination revealed a firm, well-circumscribed lesion, which was excised under local anesthesia. Histopathological analysis demonstrated large polygonal cells with granular eosinophilic cytoplasm and overlying pseudoepitheliomatous hyperplasia (PEH), while immunohistochemistry confirmed strong positivity for S-100, SOX10, and CD68, consistent with a benign GCT. No adverse histological features or malignant transformation were observed. The lesion was excised completely with narrow but clear margins, and the patient remains recurrence-free at six months of follow-up. This case highlights the importance of recognizing the characteristic histopathological and immunohistochemical features of GCT, especially in the presence of PEH, which may mimic squamous cell carcinoma. Complete excision with vigilant follow-up remains the treatment of choice, with an excellent prognosis in most cases.

## Introduction

Granular cell tumor (GCT) is a rare benign neoplasm of soft tissue, first described by Abrikossoff in 1926 as "granular cell myoblastoma" due to its granular cytoplasm and presumed muscle origin [1]. It is now understood to originate from Schwann cells (neural crest), classifying it as a peripheral nerve sheath tumor [1]. GCTs can occur throughout the body, but a significant proportion (up to 30-70%) arise in the head and neck region, most commonly on the dorsal surface of the tongue, and there is a recognized predilection for female patients [2]. Oral GCT typically presents as an asymptomatic, slow-growing, well-demarcated submucosal nodule that is firm in consistency and usually small, with the overlying mucosa generally intact [1,2]. The tumor is usually diagnosed in adulthood, with most oral cases presenting in the fourth to sixth decades of life [2]. Although histopathological and immunohistochemical features are characteristic, concurrent overlying pseudoepitheliomatous hyperplasia (PEH) may mimic squamous cell carcinoma (SCC) [3]. They are almost always benign, and malignant transformation is exceedingly rare [4]. Here we present a case of a GCT of the tongue in an adult female, detailing its clinical presentation, pathological features, and management with a brief review of the relevant literature.

## Case presentation

Patient and presentation

**Figure 1:**
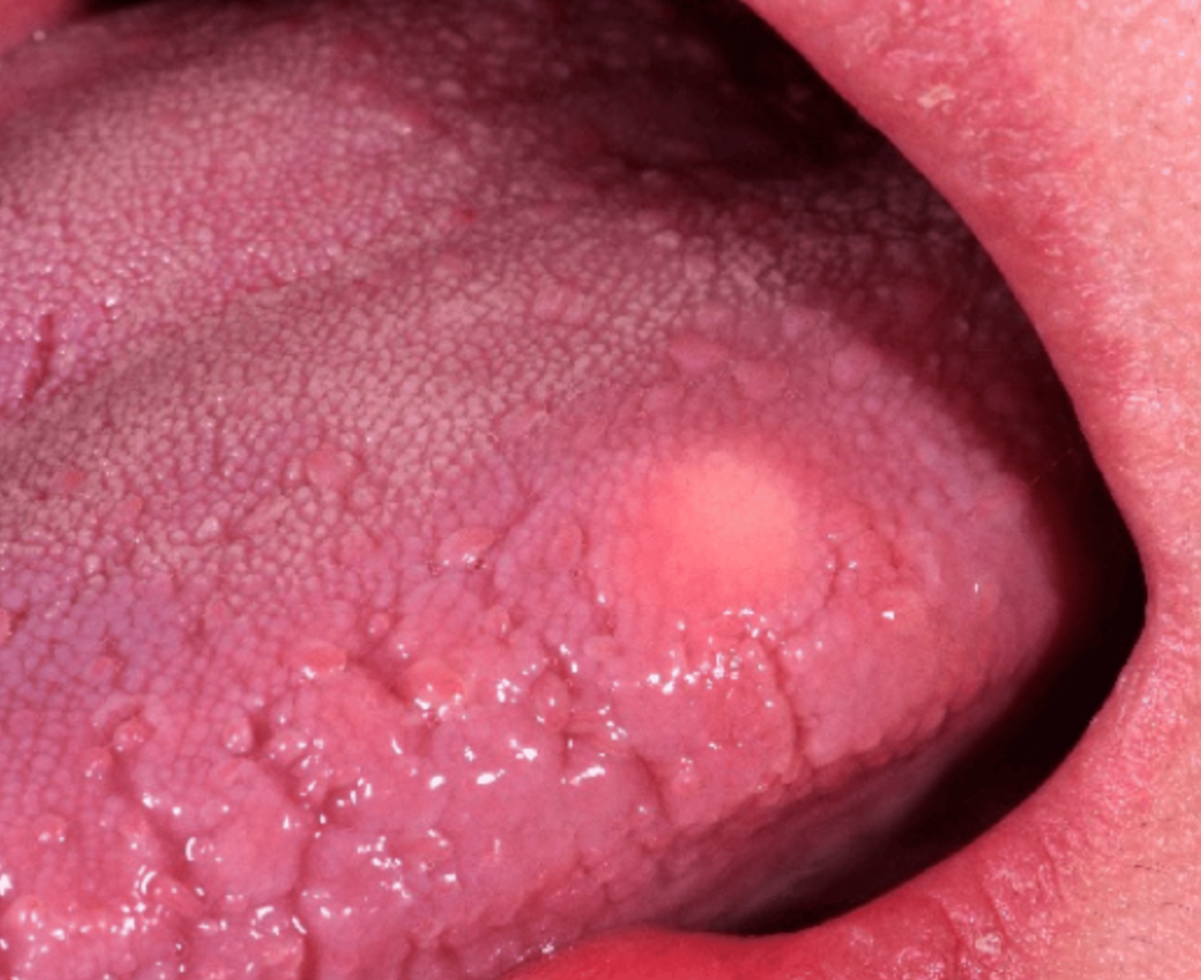
Clinical photograph showing a well-circumscribed, firm, submucosal nodule on the mid-dorsal surface of the tongue

A 58-year-old woman was referred to our clinic with a lesion on the dorsum of her tongue. She had first noticed a small “bump” on her tongue about two months prior. The lesion had grown slowly and was not causing pain, bleeding, or significant discomfort, but its persistence prompted evaluation. On oral examination, there was a single, well-circumscribed submucosal nodule on the mid-dorsum of the tongue (Figure 1). The nodule measured approximately 8 mm in greatest diameter. It was firm on palpation with smooth margins, and the overlying mucosa was intact, though slightly whitish in appearance relative to the surrounding tongue surface. No ulceration or induration of surrounding tissue was noted. There were no other lesions in the oral cavity, and the patient’s neck examination was unremarkable (no lymphadenopathy). The patient’s medical history was noncontributory, and she reported no history of trauma to the tongue.

Clinical impression and management

The differential diagnosis for an isolated, small, firm tongue nodule included benign lesions such as a fibrous GCT, neurofibroma, schwannoma, GCT, or a minor salivary gland tumor. A reactive lesion like a fibrous scar (fibroma) was also considered [2]. Given the uncertainty, an excisional biopsy was performed. The lesion was removed under local anesthesia, with an attempt to include a narrow margin of surrounding tissue. The excised specimen was a 9 mm × 5 mm piece of mucosa containing a firm submucosal nodule (~5 mm in size) with a gray-white cut surface. Healing of the tongue was uneventful, and the patient experienced minimal postoperative pain.

Histopathological findings

**Figure 2:**
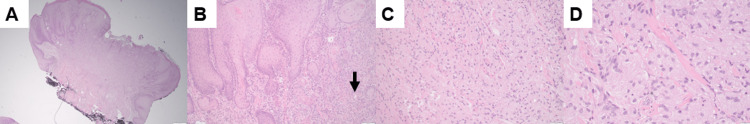
(A) Overview of the slide shows surface ulceration, pseudocarcinomatous epithelial proliferation, and a submucosal neoplasm (original magnification X20). (B) Higher magnification of the pseudocarcinomatous epithelial proliferation shows elongation of the overlying epithelium with bulbous and jagged rete ridges with occasional paradoxical maturation (arrow) and a pseudoinfiltrative pattern that can be misinterpreted as squamous cell carcinoma. However, the neoplastic granular cells can be seen between the epithelial islands on careful inspection. (original magnification X100). C. The neoplasm is composed of sheets of round and polygonal cells with abundant eosinophilic cytoplasm (original magnification X200). D. The neoplastic cells have medium-sized vesicular nuclei with small nucleoli and abundant eosinophilic cytoplasm with coarse granules (original magnification X400)

Microscopic examination of the excised tissue revealed a non-encapsulated, infiltrative submucosal tumor composed of sheets and clusters of large polygonal cells with abundant granular eosinophilic cytoplasm (Figure 2). The nuclei were generally medium-sized, round to oval, mildly pleomorphic, and occasionally contained prominent nucleoli. No significant mitotic activity was identified, and there were no areas of necrosis. The lesional cells were seen infiltrating between skeletal muscle bundles of the tongue. The overlying stratified squamous epithelium exhibited marked PEH, forming deep, bulbous, and sometimes jagged rete ridges that extended downward over the tumor nests. Despite this pseudo-carcinomatous appearance, the epithelial cells did not display dysplasia or malignancy. The histologic features were highly characteristic of a GCT. Importantly, none of the adverse histological criteria associated with malignant behavior (such as spindled cell morphology, high nucleus-to-cytoplasm ratio, brisk mitoses, or necrosis) were present in this case, indicating a benign lesion [4]. All resection margins were free of tumor, although the deep margin was very narrow (approximately 0.1 mm clear of lesional cells).

Immunohistochemistry

**Figure 3:**
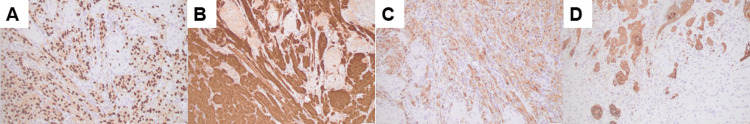
(A) The neoplastic cells express SOX10, (B) S-100, and (C) CD68. Note the negative epithelial cells of the pseudocarcinomatous proliferation. (D) The neoplastic cells are negative for AE3. The marker highlights the pseudocarcinomatous epithelial proliferation. (A-D) original magnification X200)

An immunohistochemical panel was performed to confirm the diagnosis and rule out other possibilities. The neoplastic cells showed diffuse, strong positivity for S-100 protein and for SOX10, consistent with a Schwann cell origin. There was also diffuse positivity for CD68, reflecting the rich lysosomal/granular content within the cells, which is a common feature of GCT (Figure 3). Stains for cytokeratins (AE1/AE3) and epithelial membrane antigen were negative in the neoplastic cells, excluding an epithelial lesion. However, the stains highlighted the overlying epithelial proliferation. Muscle-specific markers (desmin, smooth muscle actin, MyoD1, and myogenin) were negative, ruling out a rhabdomyomatous lesion. GATA3 was negative, excluding a paraganglioma, and neuroendocrine markers (synaptophysin, CD56) were only focally weak, findings that supported the lesion being a GCT rather than a neural/neuroendocrine tumor. The immunoprofile of S-100/SOX10 positivity with CD68 positivity is a hallmark of GCT, and, together with the histology, confirms the diagnosis of a benign GCT of the tongue.

Outcome

The patient was informed of the benign nature of the tumor. Given the clear but minimal surgical margins, she was scheduled for periodic follow-up to monitor for any signs of local recurrence. At her most recent follow-up (approximately six months post-excision), the surgical site on the tongue had entirely healed with no evidence of recurrence or new lesions. The plan is to continue clinical observation at regular intervals, as is recommended for such cases.

## Discussion

GCT of the tongue, as exemplified by this case, is an uncommon benign tumor that can pose diagnostic challenges but carries an excellent prognosis when properly managed. The tongue is the single most common site for oral GCT, and our patient’s lesion on the mid-dorsal tongue is a typical presentation [5]. The patient’s profile (middle-aged and female) also aligns with the known epidemiology of GCT, which shows a peak occurrence in adulthood (often in the fourth to sixth decade) and a female-to-male ratio around 2:1 [5]. Most GCTs are solitary; however, in roughly 10-15% of patients, multiple lesions can be present either simultaneously or metachronously in different locations [6]. No other lesions were identified in this patient, but awareness of the possibility of multifocal GCT is important for clinicians following these patients.

Histologically, GCT is characterized by large polygonal cells with abundant eosinophilic granular cytoplasm and small, uniform nuclei. The cytoplasmic granularity is due to numerous intracytoplasmic autophagolysosomes, which impart the characteristic "grainy" appearance [7]. One of the noteworthy aspects of this case is the pronounced PEH overlying the tumor. PEH is reported in a significant subset of oral GCT (ranging from about 25% to nearly 50% of cases in different series). When it is florid, it can closely simulate an SCC [3,8]. In fact, they may be initially misdiagnosed as SCC due to biopsy of only the hyperplastic epithelium or the superficial portion of the lesion [9].

Additionally, there have been cases where SCC and GCTs coexist in the same region on the tongue [10]. Fortunately, in our case, the diagnosis was made correctly by recognizing the characteristic underlying granular cells and confirming their nature with immunohistochemistry. This highlights the importance of carefully evaluating any tongue lesion with PEH for a GCT in the submucosa to avoid a case of mistaken identity. As the literature emphasizes, the epithelial hyperplasia in GCT lacks cellular atypia, and the use of immunohistochemical staining (S-100, SOX10) can definitively distinguish GCT from a true squamous neoplasm [11].

The immunohistochemical findings in this case were classic for GCT. Nearly all GCTs show strong S-100 protein positivity, reflecting their derivation from Schwann cells of peripheral nerves [12]. SOX10, a nuclear marker of Schwannian and melanocytic lineage, is similarly positive in GCT cells [12]. Our case also demonstrated CD68 positivity, which is common in GCT due to the abundance of intracytoplasmic lysosomes; however, this should not mislead one into classifying the lesion as a macrophage/histiocytic proliferation [12]. The combined immunoprofile (S-100/SOX10 positive, cytokeratin negative, etc.) is usually sufficient to confirm the diagnosis of GCT and exclude other entities. For example, the main histologic differential diagnoses include benign peripheral nerve sheath tumors like schwannoma or neurofibroma that can also be S-100 positive. However, they typically lack the distinctive granular cytoplasm and PEH seen in GCT [13]. In infants, the so-called congenital granular cell lesion (congenital epulis) superficially resembles GCT but occurs on the neonatal gingiva and is negative for S-100 protein [14]. No such confusion existed in an adult patient like ours, but it is worth noting to distinguish the entities.

Molecularly, loss-of-function mutations affecting the V-ATPase-related genes, primarily ATP6AP1 and ATP6AP2, which are implicated in endosomal regulation, or other genes involved in the endosomal/lysosomal/autophagosomal networks, are found in the majority of GCT, regardless of location or tumor behavior [15]. However, the exact mechanism by which loss-of-function mutations in endosomal regulatory proteins drive oncogenesis is currently unknown.

Complete surgical excision is the standard and curative treatment for a GCT of the tongue. Our patient’s lesion was removed fully, though with a very narrow deep margin. Lack of a well-defined capsule is common in GCT (the tumor often blends into surrounding muscle and connective tissue), so achieving a clear margin can be challenging [11]. If residual tumor cells are left behind, local recurrence can occur years later. Published recurrence rates for benign oral GCTs vary: one review noted recurrence in approximately 2-8% of cases with clear margins, rising to almost 20% when excision is incomplete [11,16]. It is reassuring, however, that even when a margin is positive, GCT tends to be slow-growing and usually remains localized [17]. For instance, a recent study of 23 tongue GCT cases found that a majority had positive deep margins on pathology, yet none showed relapse during the follow-up period [12]. In our patient’s case, although the deep margin was extremely close, no recurrence has been observed in 6 months of follow-up. We will continue to monitor our patient, and if any regrowth is detected, a prompt re-excision would be indicated. Overall, with vigilant follow-up, the prognosis for oral GCT is excellent.

Malignant GCT (MGCT) is an extraordinarily rare occurrence, and clinicians should be cautious in labeling any GCT as malignant in the absence of definitive evidence [18]. The classic Fanburg-Smith criteria (which require features such as pleomorphism, spindling, and a high mitotic rate) provide a histopathological guideline for identifying atypical or malignant cases [4]. Our case did not meet any of those criteria, which is consistent with a benign diagnosis. Even when some worrisome features are present, most such cases do not actually metastasize. In fact, some authors have argued that only the development of metastasis should define a true MGCT [19]. Thus, the emphasis is on complete excision and follow-up rather than aggressive radical treatment. In the rare event that a GCT shows multiple atypical features or frank malignancy, management may entail a wider resection and possibly adjuvant therapy. There are isolated reports of MGCT that have metastasized, and novel treatments (such as tyrosine kinase inhibitors) have been tried for unresectable cases. However, these are beyond the scope of this discussion, given our patient’s benign disease [18].

## Conclusions

GCT of the tongue is a rare benign tumor that should be included in the differential diagnosis of any persistent submucosal nodule in the oral cavity. This case highlights several key points. GCT often presents as a small, firm, asymptomatic tongue nodule in middle-aged adults, especially females. In addition, the presence of PEH over a submucosal lesion can mimic SCC, so thorough histological examination and immunohistochemical confirmation (S-100/SOX10 positivity) are crucial to avoid misdiagnosis. Finally, complete conservative excision is curative in most cases, with a low recurrence rate when adequately removed. Our patient’s outcome has been excellent, with no recurrence to date. Proper recognition of this entity is important for clinicians and pathologists, as it ensures appropriate management, avoiding overtreatment while still excising it with clear margins to prevent recurrence. With awareness of its distinctive features, GCT can be diagnosed and treated effectively, resulting in a favorable prognosis for the patient.
